# Isolated neurological presentations of mevalonate kinase deficiency

**DOI:** 10.1002/jmd2.12348

**Published:** 2022-11-18

**Authors:** Eva M. M. Hoytema van Konijnenburg, Esmeralda Oussoren, Joost Frenkel, Peter M. van Hasselt

**Affiliations:** ^1^ Department of Metabolic Diseases, Wilhelmina Children's Hospital University Medical Center Utrecht Utrecht the Netherlands; ^2^ Department of Pediatrics, Center for Lysosomal and Metabolic Diseases Erasmus University Medical Center Rotterdam the Netherlands; ^3^ Department of Pediatrics, Wilhelmina Children's Hospital University Medical Center Utrecht Utrecht the Netherlands

**Keywords:** ataxia, autoinflammation, mevalonate kinase deficiency, mevalonic acid, psychomotor delay

## Abstract

Mevalonate kinase (MK) deficiency is a rare autosomal recessive metabolic disorder caused by pathogenic variants in the *MVK* gene with a broad phenotypic spectrum including autoinflammation, developmental delay and ataxia. Typically, neurological symptoms are considered to be part of the severe end of the phenotypical spectrum and are reported to be in addition to the autoinflammatory symptoms. Here, we describe a patient with MK deficiency with severe neurological symptoms but without autoinflammation and we found several similar patients in the literature. Possibly, the non‐inflammatory phenotype is related to a specific genotype: the *MVK* p.(His20Pro)/p.(Ala334Thr) variant. There is probably an underdetection of the neurological MK deficient phenotype without inflammatory symptoms as clinicians may not test for MK deficiency when patients present with only neurological symptoms. In conclusion, although rare, neurological symptoms without hyperinflammation might be more common than expected in MK deficiency. It seems relevant to consider MK deficiency in patients with psychomotor delay and ataxia, even if there are no inflammatory symptoms.

**Key Points**

Patients with mevalonate kinase deficiency can have a severe neurological phenotype without autoinflammation. Possibly, the non‐inflammatory phenotype is related to a specific genotype: the *MVK* p.(His20Pro)/p.(Ala334Thr) variant. Consider mevalonate kinase deficiency in patients with psychomotor delay and ataxia, even if there are no inflammatory symptoms.

## 1 INTRODUCTION

Mevalonate kinase (MK) deficiency is a rare autosomal recessive metabolic disorder caused by pathogenic variants in the *MVK* gene resulting in a deficiency of the enzyme mevalonate kinase. This enzyme phosphorylates mevalonate to mevalonate 5‐phosphate, as part of the isoprenoid (including cholesterol) synthesis pathway. MK deficiency leads to the accumulation of upstream mevalonic acid and a shortage of downstream products necessary for cholesterol and nonsterol isoprenoid synthesis.^1^ The clinical spectrum of MK‐associated diseases is very broad, including autoinflammation and neurological symptoms caused by biallelic variants in the *MVK* gene, as well as the skin disorder porokeratosis, caused by dominant variants in the *MVK* gene.^2^

Patients with severe MK deficiency develop symptoms early in life and present with massive urinary excretion of mevalonic acid. Classic symptoms include autoinflammation (recurrent fever, diverse skin rashes, elevated inflammation biomarkers) with additional neurological symptoms (developmental delay, hypotonia, ataxia).^3^ Patients at the milder end of the spectrum (sometimes called hyper IgD syndrome) experience continuous or intermittent autoinflammation starting early in life, but without prominent neurological symptoms (although mood disorders and fatigue are often present). Urinary excretion of mevalonic acid is usually increased during inflammatory periods. A recent review investigating neurological symptoms of MK‐associated diseases identified only one patient who was diagnosed with MK deficiency and severe neurological symptoms who never exhibited inflammatory symptoms. The authors concluded that MK deficiency with neurological symptoms but without inflammation are extremely rare but might be underreported.^4^

We describe a patient with severe MK deficiency with neurological symptoms but without signs of inflammation and we review the literature, to illustrate the broad phenotypical spectrum of MK‐associated diseases.

## 2 CASE DESCRIPTION

The patient is a 3.5‐year‐old girl, born after an uneventful pregnancy and term birth. In the first year of life, she suffered from several upper respiratory tract infections for which she received antibiotics, always with good effect within one day. She underwent an adenotonsillectomy at age 14 months, whereafter the upper airway infections stopped. She had one urinary tract infection. She was referred to a pediatrician at age 2 years as she could not walk independently. On physical evaluation, there was a psychomotor delay and ataxia, for which a magnetic resonance imaging was made at age 2 years 4 months which showed cerebellar atrophy. A metabolic screening targeting developmental delay revealed massively increased urinary mevalonic acid of 3243 mmol/mol creatinine (reference range 0.1–0.7) after several months increased to 5085.8 mmol/mol creatinine. The diagnosis of MK deficiency was confirmed by measurement of mevalonate kinase enzyme activity which was below 2 pmol/min/mg protein (reference range 125–395) and by genetic testing showing compound heterozygous variants in the *MVK* gene (c.59A > C (p.(His20Pro)) and c.1000G > A (p.(Ala334Thr))). She was referred to a neurologist, a metabolic pediatrician and an ophthalmologist for monitoring. At age 3 years 3 months, she cannot walk without a walking frame, and she is severely ataxic, which seems to be aggravated during infections. Her cognitive development is within normal range. Eye examination shows a refractive error, but no retinal anomalies.

She never had periodic fevers or fevers of unknown origin, infections never lasted longer than expected, and there were never any skin rashes or other associated symptoms with fever other than the obvious source of infection. According to her parents, she is rarely ill and she is not different from her two older (healthy) siblings in this regard.

### 2.1 Discussion

Inflammatory symptoms are the hallmark of MK deficiency in nearly all patients, with additional neurological symptoms in some. In a recent systematic review,^4^ over 300 patients were included (292 from cohort studies, 45 from case studies, although possibly with some overlap) of which the majority experienced autoinflammation. One patient with severe neurological symptoms (intellectual disability, ataxia and ocular symptoms) without inflammation is described.^5^ Furthermore, one patient who presented with retinal dystrophy experienced no other neurological or inflammation symptoms^6^; and for two other siblings, with milder neurological symptoms, it was not completely clear if there had been inflammatory symptoms.^7^, ^8^ Aside from the patients included in this review, we found two other case‐descriptions about MK deficient patients with neurological symptoms without autoinflammation: one describing two siblings presenting with developmental delay, cerebellar atrophy with ataxia and hypotonia^9^ and another describing a patient with developmental delay, cerebellar atrophy and retinal dystrophy.^10^

Here, we describe another patient who has MK deficiency with severe and typical neurological symptoms but no autoinflammation. Although it is possible that inflammatory symptoms could arise later in life, periodic fever episodes usually start very young and tend to lessen with age.^8^ Autoinflammation in MK deficiency is driven by defective prenylation due to downstream reduced isoprenoid synthesis which results in reduced inflammasome inhibition, ultimately leading to excessive interleukin 1 (IL‐1, a potent pro‐inflammatory cytokine) secretion.^2^, ^11^ Following this, IL‐1 blocking therapy is the standard of care treatment for auto‐inflammatory symptoms in MK deficiency, either continuously or intermittently at the onset of flares.^12^ Although the exact pathophysiological mechanism of neurological manifestations of MK deficiency is unknown, it is proposed that, on the one hand, downstream prenylation defects cause central nerve system (CNS) inflammation and neurodegeneration. On the other hand, shortage of CNS cholesterol causes myelination defects and neuronal impairments.^4^ In addition, upstream accumulation of mevalonic acid may also be toxic for the CNS.^2^ Statin therapy (which blocks HMG‐CoA reductase upstream of MK, thus reducing mevalonic acid levels) has been tried in some patients, with mixed but overall disappointing results and, in some patients, even aggravation of auto‐inflammation, although the effect on neurological symptoms is unknown.^13^ Cholesterol supplementation has been tried and was unsuccessful as well.^14^ While IL‐1 blocking therapy is successful in controlling autoinflammatory symptoms, currently, no treatment for neurological symptoms in MK deficiency exists. Only anecdotally, improvement of neurological symptoms after allogeneic stem cell transplantation has been reported, but with significant transplantation‐related morbidity and mortality.^15^

Since it is thought that both the auto‐inflammation and neurological symptoms are mostly caused by downstream effects of the MK deficiency, and the vast majority of patients experience only inflammation symptoms, with or without neurological symptoms, a patient with only severe neurological symptoms does not fit the clinical vignette. It cannot be fully excluded that what was reported as normal childhood infections in our patient may, in fact, have been auto‐inflammatory attacks. However, the attacks of MK deficiency are stereotypical, that is, patients and parents recognize them as the very same thing as all previous attacks. The attacks are accompanied by specific tissue involvement, as reflected in the 2019 Eurofever classification criteria for auto‐inflammatory recurrent fevers. Classification criteria for autoinflammation in MK deficiency are evidence of elevation of acute phase reactants in correspondence to the clinical flares with recurrent disease activity of at least 6 months with an MK deficiency genotype and gastrointestinal symptoms or cervical lymphadenitis or aphthous stomatitis.^16^ Up until now, our patient does not meet these criteria at all.

Interestingly, the only other patient from the systematic review with severe neurological symptoms but no inflammatory symptoms has the same genotype as our patient (H20P/A334T),^5^ so a genotype–phenotype correlation might be considered. The symptoms of that patient started at 12 months of age, and this case was reported at 54 years. The patient experienced intellectual disability, ataxia, cataract and retinal degeneration. Urinary mevalonic acid concentration was 2730 mmol/mol creatinine and MK enzyme activity was 1.1%. In addition to this patient, there are three other patients with the H20P/A334T genotype reported in the literature, according to a recent review on genotype–phenotype correlation.^3^ The first patient (who was the first patient ever reported with increased urine mevalonic acid^17^) experienced moderate ataxia and severe cerebellar atrophy. Measurements of enzyme activity in fibroblasts showed virtually no activity. Recurrent febrile crises are mentioned in some reports but not in all. At age 20, he was reported to be in a good clinical condition,^18^ at age 41 he was reported to use canakinumab therapy.^8^ Furthermore, two siblings with the H20P/A334T phenotype are reported, who presented with neurological symptoms (ataxia, retinal dystrophy and, in one sibling, mild retardation noticed after meningitis at the age of 12 years). Information on hyperinflammation is contradicting between reports, but for these siblings also, inflammation is not an important part of the phenotype.^7^, ^8^

Overall, it seems that for most reported patients with the *MVK* H20P/A334T genotype, inflammation is not a major symptom. Looking at the genotype of the other reported patients with neurological symptoms without inflammation: one has the A334T variant with a different second variant^10^; one has two A334T variants^6^; and for two siblings, the genotype is not reported.^9^ According to a recent review, the A334T variant is, either homozygous or compound heterozygous with H20P, associated with severe ocular and neurological symptoms.^3^ Apparently, autoinflammation is not always associated.

\One possible factor in explaining phenotypical differences between patients is the influence of other genes (second hit or modifier genes) in addition to the known *MVK* gene variants. An example has been reported in a multi‐omics study of two siblings with biochemical signs of MK deficiency and the same genotype of known pathogenic variants in the *MVK* gene, but with a radically different phenotype: one sibling experienced auto‐inflammatory symptoms while the other sibling was asymptomatic.^19^ The symptomatic sibling only was found to have a missense variant in the STAT1 gene, in addition to the pathogenic variants in the *MVK* gene, leading to increased activation of the Janus kinase/signal transducer and activator of transcription proteins (JAK/STAT) signaling pathway, an inflammatory pathway that is thought to have a synergistic effect with the MK deficiency and explain the auto‐inflammation in the symptomatic sibling.^19^ It is conceivable that a similar phenomenon is present in other patients as well and might explain some of the differences in the presence of autoinflammation between patients.

Finally, it is probable that MK deficiency without inflammatory symptoms is under‐reported as clinicians may not test for MK deficiency in patients with only neurological symptoms. For our patient, urinary mevalonic acid measurement was part of a broad screening for developmental delay and ataxia, but this may not always be the case.

In conclusion, we describe a patient with MK deficiency with severe neurological symptoms but without autoinflammation and we found several other patients in the literature. Although rare, it might be more common than expected and seems relevant to consider MK deficiency in patients with psychomotor delay and ataxia, even if there are no inflammatory symptoms.

## AUTHOR CONTRIBUTIONS

Eva MM Hoytema van Konijnenburg was involved in conception/design of the study, performing the literature search and drafting the article. Peter M. van Hasselt was involved in the design of the study, interpretation of the data and revising the article. Joost Frenkel was involved in the design of the study, interpretation of the data and revising the article. Esmeralda Oussoren was involved in interpretation of the data and revising the article.

## FUNDING INFORMATION

There was no specific funding for this study.

## CONFLICT OF INTEREST

Eva MM Hoytema van Konijnenburg, Esmeralda Oussoren, Joost Frenkel and Peter M. van Hasselt declare that they have no conflict of interest.

## ETHICS STATEMENT

Ethics approval was not required for this study.

## INFORMED CONSENT

All procedures followed were in accordance with the ethical standards of the responsible committee on human experimentation (institutional and national) and with the Helsinki Declaration of 1975, as revised in 2000. Additional informed consent was obtained from the parents of the patient for whom identifying information is included in this article and is available upon request.

## TAKE‐HOME MESSAGE

Consider mevalonate kinase deficiency in patients with psychomotor delay and ataxia, even if there are no inflammatory symptoms.

## Data Availability

The data that support the findings of this study are available from the corresponding author (EHvK), upon reasonable request.
